# Declines in Pneumonia Mortality Following the Introduction of Pneumococcal Conjugate Vaccines in Latin American and Caribbean Countries

**DOI:** 10.1093/cid/ciaa614

**Published:** 2020-05-25

**Authors:** Lucia H de Oliveira, Kayoko Shioda, Maria Tereza Valenzuela, Cara B Janusz, Analía Rearte, Alyssa N Sbarra, Joshua L Warren, Cristiana M Toscano, Daniel M Weinberger, Multinational Study for PCV Impact in Mortality Study Team

**Affiliations:** 1 Comprehensive Family Immunization Unit, Family, Health Promotion, and Life Course, Pan American Health Organization, World Health Organization, Washington, District of Columbia, USA; 2 Department of Epidemiology of Microbial Diseases, Yale School of Public Health, New Haven, Connecticut, USA; 3 Department of Public Health and Epidemiology, Universidad de Los Andes, Santiago, Chile; 4 School of Medicine, Universidad Nacional de Mar del Plata, Mar del Plata, Province of Buenos Aires, Argentina; 5 Department of Biostatistics, Yale School of Public Health, New Haven, Connecticut, USA; 6 Department of Collective Health, Institute of Tropical Pathology and Public Health, Federal University of Goias, Goiânia, Goiás, Brazil

**Keywords:** pneumococcal conjugate vaccines, vaccine evaluation, pneumonia, childhood mortality, Latin America and Caribbean

## Abstract

**Background:**

Pneumococcal conjugate vaccines (PCVs) are recommended for use in pediatric immunization programs worldwide. Few data are available on their effect against mortality. We present a multicountry evaluation of the population-level impact of PCVs against death due to pneumonia in children < 5 years of age.

**Methods:**

We obtained national-level mortality data between 2000 and 2016 from 10 Latin American and Caribbean countries, using the standardized protocol. Time series models were used to evaluate the decline in all-cause pneumonia deaths during the postvaccination period while controlling for unrelated temporal trends using control causes of death.

**Results:**

The estimated declines in pneumonia mortality following the introduction of PCVs ranged from 11% to 35% among children aged 2–59 months in 5 countries: Colombia (24% [95% credible interval {CrI}, 3%–35%]), Ecuador (25% [95% CrI, 4%–41%]), Mexico (11% [95% CrI, 3%–18%]), Nicaragua (19% [95% CrI, 0–34%]), and Peru (35% [95% CrI, 20%–47%]). In Argentina, Brazil, and the Dominican Republic, the declines were not detected in the aggregated age group but were detected in certain age strata. In Guyana and Honduras, the estimates had large uncertainty, and no declines were detected. Across the 10 countries, most of which have low to moderate incidence of pneumonia mortality, PCVs have prevented nearly 4500 all-cause pneumonia deaths in children 2–59 months since introduction.

**Conclusions:**

Although the data quality was variable between countries, and the patterns varied across countries and age groups, the balance of evidence suggests that mortality due to all-cause pneumonia in children declined after PCV introduction. The impact could be greater in populations with a higher prevaccine burden of pneumonia.

## Teaser

The introduction of pneumococcal conjugate vaccines is associated with substantial declines in childhood pneumonia mortality. Approximately 4500 pneumonia deaths were estimated to have been averted among children aged 2–59 months across 10 Latin America and Caribbean countries since vaccine introduction.

Pneumococcal infections are one of the primary causes of illness and death in children < 5 years of age worldwide [1]. Pneumococcus causes a variety of syndromes, including pneumonia, meningitis, and bacteremia, as well as milder but more common illnesses such as otitis media and sinusitis [2]. Prior to the introduction of pneumococcal conjugate vaccines (PCVs), pneumococcus caused about 600 000 deaths globally in children aged 1–59 months in 2000 [3].

PCVs have now been introduced in > 140 countries, including in many low- and middle-income settings [4]. In 2012, the World Health Organization recommended introduction of PCVs into national routine immunization programs (NIPs) worldwide, with high priority to countries with a high childhood mortality rate [5]. Their latest recommendation reinforces the recommendation, regardless of disease and mortality burden levels [6].

As PCVs are adopted in additional countries, it is critical to document the effect of their introduction. This information is important for decision-makers at the country level and for international partners and donors. PCVs have a well-documented impact against morbidity due to bacteremic pneumococcal disease, meningitis, otitis media, and pneumonia [7–9]. However, many decision-makers are strongly interested in the ability of PCVs to prevent deaths.

Evaluating the impact of PCVs on mortality due to pneumonia is challenging because pneumococcus is just one of many pathogens that can cause pneumonia. Therefore the relative decline in disease rates due to the vaccine might be modest. Most of the studies that have attempted to evaluate the impact of PCVs against pneumonia mortality have been conducted in Latin America and Caribbean (LAC) countries [10–14]. These studies have yielded inconsistent results, using different methodologies, and the estimates could differ based on the socioeconomic characteristics of the location [14]. This highlights the need for high-quality studies that evaluate the impact of PCVs against pneumonia mortality across countries using standardized data extraction and analytic methods.

In this study, we estimated declines in mortality due to all-cause pneumonia in 10 LAC countries. LAC countries were at the forefront of PCV introduction globally, with many having introduced the vaccine in the last decade [15]. LAC countries make an ideal study setting for measuring the impact of PCVs because they represent a wide range of socioeconomic levels and have used various commercially available vaccines (7-valent, 10-valent, and 13-valent PCVs) with a variety of dosing strategies (3 + 1, 2 + 1, and 3 + 0 schedules). The LAC region is also unique in having relatively high-quality death registries in most countries [16]. This multicountry study aims to provide the most comprehensive evidence to date on changes in mortality due to all-cause pneumonia associated with the universal introduction of PCVs into NIPs, using standardized methodology.

## METHODS

### Mortality Data

We selected 10 LAC countries with extensive experience using PCV in their NIPs based on the inclusion criteria (Supplementary Data). Of these, 3 countries are eligible for Gavi support (Guyana, Honduras, Nicaragua), and 7 countries are currently not eligible for Gavi support (Argentina, Brazil, Colombia, the Dominican Republic, Ecuador, Mexico, and Peru).

We used mortality data from national-level mortality registries, after the countries conducted standardized data cleaning and quality control [17]. Mortality data for children aged < 5 years available during the study period (2005–2016) were extracted from the National Mortality Information Systems in each country. For each death record, we extracted the month and year of birth and death, age of the individual at death, and *International Classification of Diseases, Tenth Revision* (*ICD-10*) codes. Three immunization periods were considered for each country, depending on the year of universal introduction of PCVs: (1) prevaccine period; (2) transition period, which is the first 12 months after universal introduction of PCV in the country to allow vaccine uptake to stabilize; and (3) evaluation period, when PCV coverage had reached a stable level among infants.

To assess data quality, we obtained standardized indicators for each country, including the proportions of underreporting, completeness of death registration with cause of death, reported deaths with “ill-defined” causes of death (*ICD-10* codes R00–R99), and the proportion of deaths in which “garbage codes” are indicated as causes of death [18].

Data were also stratified by the following age groups: 2–11, 12–23, and 24–59 months. Children aged < 2 months were excluded from the analyses for multiple reasons (Supplementary Figure 1).

### Statistical Analysis

The intervention of interest was introduction of PCV into the NIP, regardless of the vaccine product, schedule, or use of catch-up strategy (Supplementary Table 1). The outcome of interest was death due to all-cause pneumonia, defined as having an *ICD-10* code for main/underlying cause of death in the range of J12–J18. Cause-specific codes (eg, J13: pneumococcal pneumonia) were used too rarely to be useful by themselves for analysis purposes. Some countries recorded multiple causes of death, and we performed sensitivity analyses in which we counted a code of J12–J18 in any of the diagnostic fields as a death due to pneumonia.

The goal for these analyses was to estimate declines in childhood mortality due to all-cause pneumonia following the introduction of PCV. To obtain robust estimates of the decline, it is crucial to isolate changes in pneumonia mortality caused by PCV from those caused by other factors, such as underlying health of the population and reporting. To do this, we used “synthetic control (SC)” models as our primary analysis for most subgroups and the “seasonal-trend decomposition plus principal component analysis (STL + PCA)” models when the data were sparse [19, 20]. The advantages of SC and STL + PCA models are as follows: (1) They are more likely to correctly adjust for these unmeasured confounding and biases than other methods (eg, interrupted time series) by using information on control conditions that were affected by the same/similar unrelated factors; (2) they either automatically select an appropriate set of control conditions or create a consensus trend based on the prevaccine data, without requiring users to handpick them a priori; and (3) there is no need to make an assumption that trends in the prevaccine period linearly continue into the postvaccine period. Their limitation is that they cannot adjust for underlying trends specifically associated with all-cause pneumonia deaths (eg, changes in treatment practices for respiratory diseases or declines in the use of indoor cookstoves). However, if we can assume that treatment practices for any kind of diseases are in general improving over time, those trends are captured by other control causes of deaths in these models.

In most instances, we used the SC model, which is a Poisson regression model where the outcome is the number of all-cause pneumonia deaths per month (or quarter for Guyana due to sparse counts at the monthly time scale), and the covariates are time series for other categories of death that are not likely to be influenced by PCVs (Supplementary Table 2) [19]. The SC model uses a Bayesian variable selection method to give more weight to control causes of death that are most correlated with pneumonia deaths in the prevaccine period. This effectively creates a composite of control causes of death against which pneumonia deaths are compared in the evaluation period. To quantify the impact of PCV, we calculated a rate ratio (RR), which is the number of observed pneumonia deaths divided by the expected number of pneumonia deaths if intervention had not been introduced for the evaluation period (the counterfactual).

Although the SC approach is a powerful tool to estimate the impact of interventions, it fails to identify an appropriate set of control conditions when data are sparse [20]. In such settings, we used the “STL + PCA” approach [20]. In this method, instead of using a large number of control causes of death, we used a single consensus trend of the presmoothed versions of the control causes of death and used this in the Poisson regression model to adjust for unrelated changes in pneumonia deaths. This alternative approach enabled us to keep strengths of the SC method, while solving issues introduced by sparse data. More details for these models can be found in the Supplementary Data.

As a sensitivity analysis, we also performed a simple analysis where we adjusted only for all nonrespiratory deaths and for linear trends to control for unrelated changes. The results of this time trend adjustment are presented alongside the results from the SC models and STL + PCA models for comparison.

All analyses were performed in R (Vienna, Austria). The aggregated time series data and code can be found in the following github repository: https://github.com/weinbergerlab/paho-pneumonia-mortality.

### Ethical Considerations

The study protocol was reviewed and approved by institutional review boards in each country and also by the Pan American Health Organization (PAHO) Ethical Research Committee.

## RESULTS

### Descriptive Results

We obtained mortality data from 10 LAC countries. Countries had 4–11 years of prevaccine data and 2.3–8.9 years of postvaccine data. The reported third dose coverage ranged from 81% to 100% at the national level in all countries except for the Dominican Republic (Supplementary Table 1). The Dominican Republic prioritized administration of 2 doses due to a short-term shortage of vaccine supply, so it had low uptake of 3 doses.

**Figure 1.**
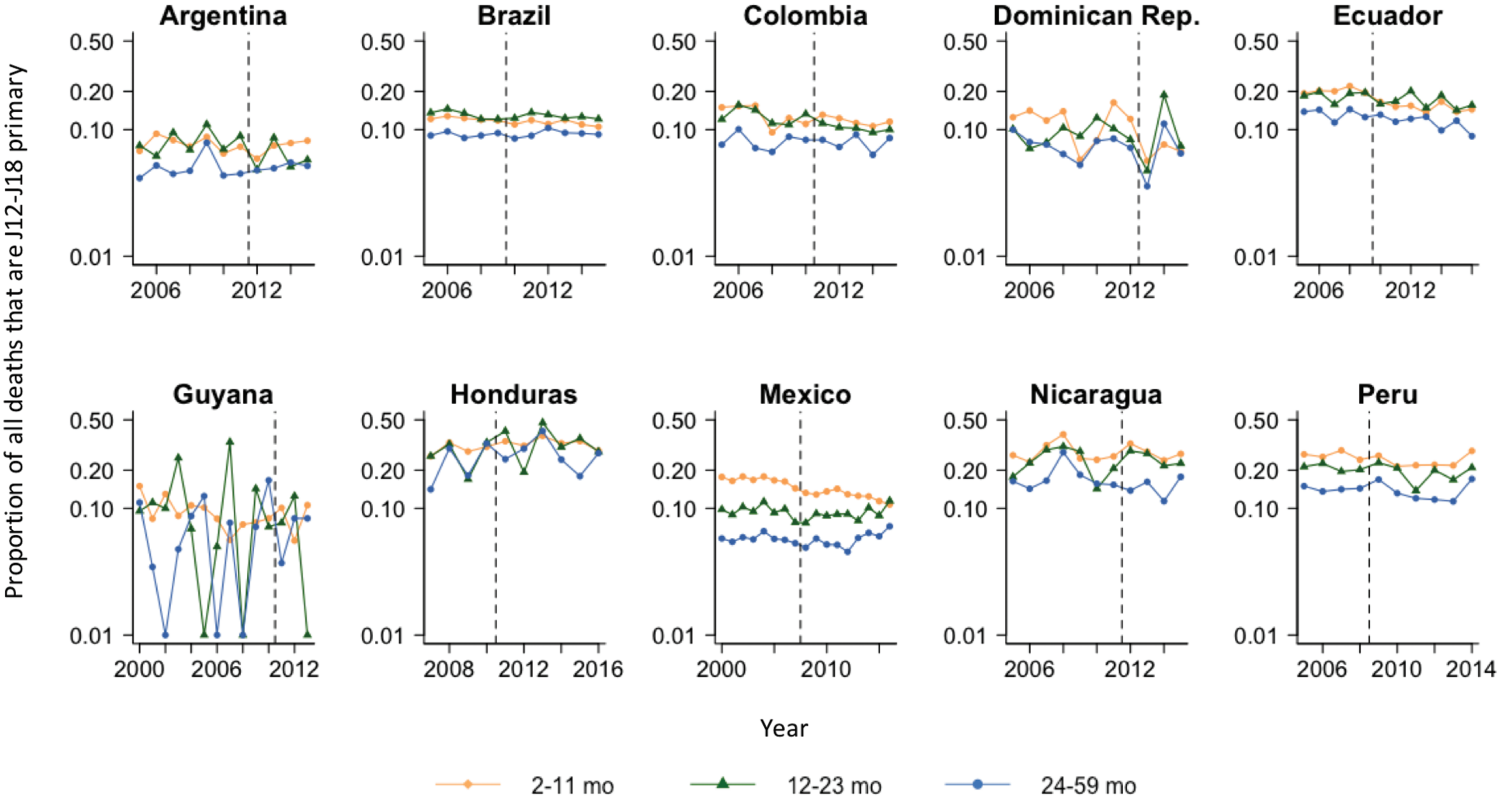
Annual time series for the proportion of all deaths that are caused by all-cause pneumonia (*International Classification of Diseases, Tenth Revision* [*ICD-10*] codes J12–J18 as primary cause of death) by age group in 10 Latin American and Caribbean countries. Vertical dashed lines represent the timing of pneumococcal conjugate vaccine introduction. The y-axis is on the log scale. For Guyana, the proportion was 0 in some years, which was replaced with 0.01 to allow for log transformation.

In total, there were 73 912 deaths due to all-cause pneumonia among children aged 2–59 months during the study period. The reported incidence of death due to all-cause pneumonia per 100 000 among children aged 2–59 months in the pre-PCV period ranged from 7.8 in Argentina to 29.6 in Peru (Supplementary Table 1). The reported incidence from the Dominican Republic was low, which reflects the low reporting rates for the country. The fraction of all deaths attributed to all-cause pneumonia varied by country and age group (Figure 1).

Standardized data quality indicators varied significantly by country and within a given country over time (Supplementary Figure 2). Quality of mortality data seems to have improved over time in most countries, as demonstrated by the declining trends in percentage of reported deaths with ill-defined causes of death, and declining trends for percentage of deaths with garbage codes in most countries. While some countries still present with a high proportion of underregistered deaths, namely the Dominican Republic and Peru, this indicator has also been declining in the most recent years in most countries.

### Declines in All-cause Pneumonia Mortality Among Children Aged 2–59 Months

**Figure 2.**
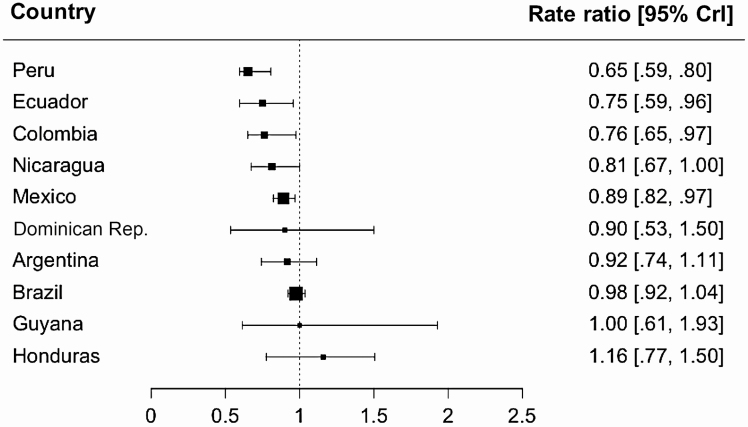
Estimated impact of pneumococcal conjugate vaccine among children aged 2–59 months in 10 Latin American and Caribbean countries. Rate ratios were calculated by dividing the cumulative number of observed pneumonia deaths by the cumulative number of predicted pneumonia deaths during the evaluation period. Black squares represent the point estimates of rate ratio and bars represent their 95% credible intervals. The size of squares is proportional to the average number of pneumonia deaths per year, which is a proxy of sample size. Abbreviation: CrI, credible interval.

**Figure 3.**
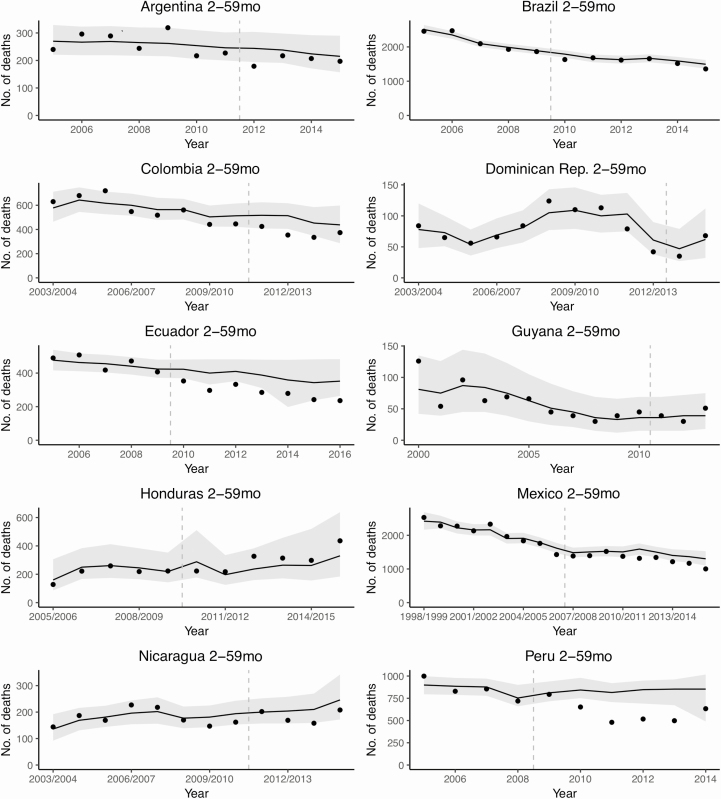
Annual time series for the observed and predicted number of pneumonia deaths among children aged 2–59 months in 10 Latin American and Caribbean countries. Dots represent the observed number of pneumonia deaths (*International Classification of Diseases, Tenth Revision* codes J12–J18). Lines and gray-shaded areas represent point estimates and 95% credible intervals of the predicted pneumonia deaths, respectively. Vertical dashed lines show the timing of pneumococcal conjugate vaccine introduction in each country. Year was defined as January–December in the countries in the Southern Hemisphere, and July–June in the countries in the Northern Hemisphere.

Many countries showed some evidence of a decline in mortality due to all-cause pneumonia among children aged 2–59 months following the introduction of PCV, after adjusting for underlying trends (Table 1 and Figures 2 and 3). The estimated decline was strongest in Colombia, Ecuador, Mexico, Nicaragua, and Peru, where the estimated RRs ranged from 0.65 to 0.89. There was a high level of uncertainty in the estimated RRs in Guyana, the Dominican Republic, and Honduras. In contrast, the estimated RRs in Argentina and Brazil were close to 1 (no detectable decline), with a moderate level of uncertainty. The estimates of RRs were somewhat sensitive to the choice of analysis model, but the overall pattern across countries remained the same (Supplementary Table 3). The top 3 control causes of death selected by the Bayesian variable selection method in the SC model are shown in Supplementary Table 4. As a sensitivity analysis, we ran the SC models after excluding these top 3 control diseases, and found that estimated RRs did not change substantially (Supplementary Table 4). Across the 10 countries, PCV introduction was associated with a reduction of 4500 pneumonia deaths among children aged 2–59 months during the postvaccine period.

**Table 1.** Estimated Impact of Pneumococcal Conjugate Vaccine and Deaths Averted Among Children 2–59 Months of Age in 10 Latin American Countries

Country	Postvaccine Period	Estimated Rate Ratio (95% CrI)^a^	Estimated Total Deaths Averted Since PCV Introduction (95% CrI)	Average Estimated Total Deaths Averted per 100 000 Population per Year in the Postvaccine Period (95% CrI)^b^
Argentina	Jan 2012 to Dec 2015	0.92 (.74–1.11)	122 (−26 to 313)	0.8 (−.2 to 2.2)
Brazil	Mar 2010 to Dec 2015	0.98 (.92–1.04)	390 (−138 to 921)	0.4 (−.1 to .9)
Colombia	Nov 2011 to Dec 2015	0.76 (.65–.97)	476 (45–789)	2.7 (.3–4.6)
Dominican Republic	Sep 2013 to Dec 2015	0.9 (.53–1.5)	22 (−21 to 87)	1 (−.9 to 3.8)
Ecuador	Aug 2010 to Dec 2016	0.75 (.59–.96)	597 (117–1168)	5.7 (1.1–11.2)
Guyana	Jan 2011 to Dec 2013	1 (.61–1.93)	−1 (−17 to 21)	−0.5 (−8.3 to 10.3)
Honduras	Jan 2011 to Dec 2016	1.16 (.77–1.5)	−141 (−482 to 548)	−2.8 (−9.5 to 10.8)
Mexico	Feb 2008 to Dec 2016	0.89 (.82–.97)	1426 (439–2433)	1.5 (.5–2.5)
Nicaragua	Jan 2012 to Dec 2015	0.81 (.67–1)	117 (−22 to 274)	4.4 (−.8 to 10.3)
Peru	Aug 2009 to Dec 2014	0.65 (.59–.8)	1453 (743–1831)	9.5 (4.9–12)

Abbreviations: CrI, credible interval; PCV, pneumococcal conjugate vaccine.

^a^Estimated rate ratios were calculated by dividing the cumulative number of observed pneumonia deaths by the cumulative number of predicted pneumonia deaths during the evaluation period.

^b^Details of the calculation of estimated total deaths averted per 100 000 population per year can be found in the Supplementary Data.

### Variations in the Decline in All-cause Pneumonia Mortality by Age Group

**Figure 4.**
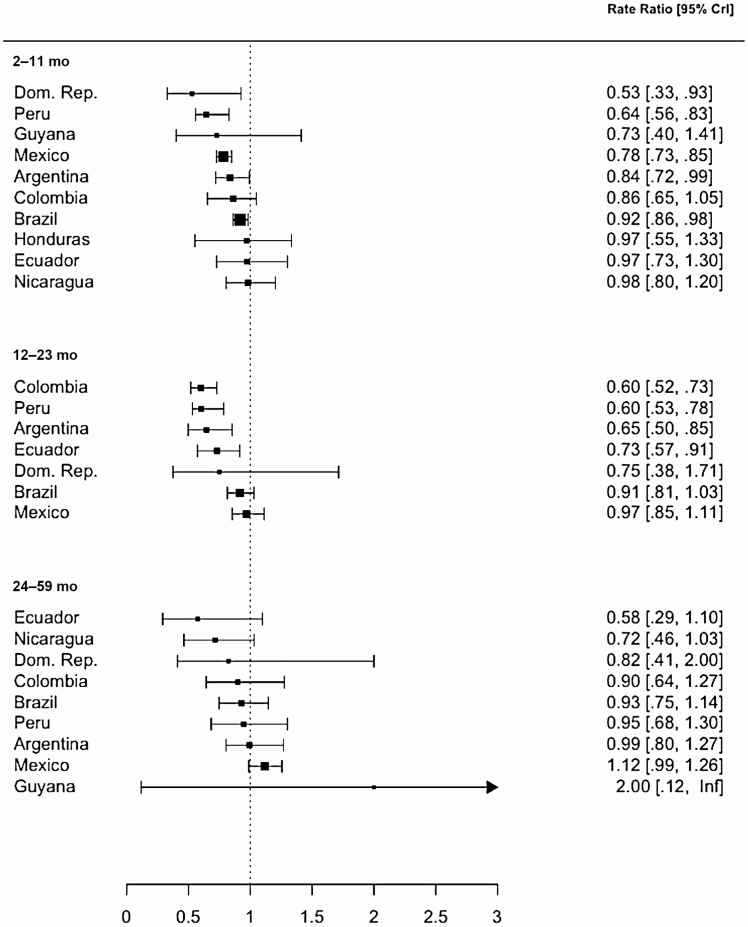
Estimated impact of pneumococcal conjugate vaccine by age group in 10 Latin American and Caribbean countries. Rate ratios were calculated by dividing the cumulative number of observed pneumonia deaths by the cumulative number of counterfactual pneumonia deaths during the evaluation period. Black squares represent the point estimates of rate ratio and bars represent their 95% credible intervals (CrIs). The size of squares is proportional to the average number of pneumonia deaths per year, which is a proxy of sample size. Some countries are not included in certain age groups, as their data were very sparse and we could not generate reliable estimates. An upper bound of the 95% CrI for 24–59 months of age in Guyana was infinity, because the denominator of the rate ratio was zero. Abbreviations: CrI, credible interval; Dom. Rep., Dominican Republic; Inf, infinity.

The estimated decline in mortality due to all-cause pneumonia differed by age group for which we were able to obtain estimates (Figure 4, Supplementary Figure 3, Supplementary Table 5). Among children aged 2–11 months, strong declines were apparent in the Dominican Republic, Mexico, and Peru, and smaller declines were evident in Argentina and Brazil. In contrast, among children aged 12–23 months, there was strong evidence for declines in Argentina, Colombia, Ecuador, and Peru, and weak evidence of declines in Brazil. Among children aged 24–59 months, declines were not evident in any countries, potentially due to higher uncertainty in these estimates.

### Sensitivity Analyses

We performed a number of sensitivity analyses. These included using different modeling approaches, excluding specific control variables from the SC analysis, using a definition of all-cause pneumonia that searched for the relevant *ICD-10* codes anywhere in the record (rather than the primary cause), and using the 2-year transition period (Supplementary Tables 3, 4, 6, and 7). Some of these options influenced the magnitude of the estimates or whether or not the 95% credible intervals (CrIs) included one; however, the overall conclusions from the analyses were generally the same. In Peru, the estimated RR changed from 0.65 (95% CrI, .59–.8) to 0.98 (95% CrI, .84–1.15) after using all-cause pneumonia coded anywhere in the record as the outcome as opposed to coded only for primary cause. This is likely because coding practices changed over time, and codes J12–J18 was less commonly coded as the primary cause of deaths in Peru.

## Discussion

Using a comprehensive set of death records from 10 LAC countries, we demonstrate that the introduction of PCVs coincided with declines in all-cause pneumonia mortality in many countries. The quality of the data was variable between countries, and the estimates were somewhat sensitive to the analysis approach. As a result, the patterns were complex and varied across countries and age groups. The balance of evidence, however, suggests that the observed declines were due to PCVs. As most countries in our study have low to moderate incidence of pneumonia mortality, the benefit of PCVs could be much greater if the vaccine is introduced into a setting with higher burden.

The variation in the impact of PCV across countries could have various explanations. First, although the coding of the causes of death was thoroughly reviewed and cleaned by the Ministry of Health and PAHO using the standardized data cleaning and quality control methods, several aspects of quality may lead to over- or underestimates of the impact. The countries varied tremendously in the completeness of death reporting, and this could change over time. Moreover, the cause of death in many settings was not reported consistently, and there was an overuse of the codes for “unspecified” cause of death. These variations in data quality across countries and within a given country over time were controlled for to some extent in the SC and STL + PCA models, as these variations affected both our outcome and control causes of death. Nevertheless, it should be noted that data quality influences the robustness of results, and results should be interpreted accordingly. Second, the duration of postvaccine data differed across countries, and estimates in countries with longer postvaccine data are more likely to achieve full coverage and also to be influenced by serotype replacement [21]. Third, in Guyana and Honduras, there was a high level of uncertainty in the estimates, and evidence of a decline associated with the introduction of PCVs was not detectable in any of the age groups. As deaths are a relatively infrequent event, there is a high degree of unexplained variability in the data, which leads to wide uncertainty intervals. Further work is needed to determine whether the lack of a detectable decline is due to excess variability in the data (low power) or whether this represents a true lack of an effect.

The estimated impact of PCVs also varied across age groups. Some countries observed declines among children 2–11 months old and those 12–23 months old, whereas others only detected declines among one of these groups; this might be due to the different disease burden of pneumococcus across age groups. For example, various pathogens cause pneumonia deaths among children 2–11 months old in some countries, and as a result, only a small or undetectable decline was observed following the introduction of PCV.

A number of approaches are commonly employed to evaluate vaccine impact [19, 20]. These methods differ in how they adjust for trends unrelated to vaccination and seasonality and how they quantify the vaccine-associated changes. The different approaches have different assumptions about the underlying trends in the data. Because there is not a ground-truth estimate of the impact of PCVs on pneumonia mortality against which we can compare these estimates, there is no way to definitively determine which method is correct. However, we have found in most settings that the SC model outperforms other methods in adjusting for underlying trends in the data, except when the control time series are very sparse [20]. As a comparison, we also fit a simple model that adjusts for linear trends and all-cause mortality, and the STL + PCA model, which can help to adjust for trends when the control time series are sparse [20]. By including the results from multiple methods in the supplement, we hope to highlight settings where there is more or less consensus among the methods.

While numerous studies have demonstrated the effects of PCVs against invasive pneumococcal disease and morbidity due to pneumonia, limited real-world evidence of the impact of PCVs on mortality has been available (see review of previous literature in the Supplementary Data). Many of these studies used time series analyses that were unable to control for unrelated secular trends in deaths and had little prevaccine data. While previous studies provide qualitatively similar findings to our results, our study is the most comprehensive, multinational study and provides the strongest evidence of the real-world impact of PCVs against pneumonia mortality in children. One of the strengths of our analyses lies in having relatively long prevaccine baseline periods, which enabled us to adjust for trends unrelated to the vaccine. Also, standardized methodology for data extraction and analysis made the estimates across the countries more comparable. We also conducted rigorous evaluation of the sensitivity of the results to various assumptions.

In conclusion, we have evaluated the impact of PCV using routinely collected data from national mortality registries in the LAC region. This provides an important proof of concept for using similar methods to evaluate vaccine impact in other countries around the world. Such assessments are crucial for decision-makers at various levels of responsibility. Our results demonstrate substantial reductions in mortality due to pneumonia in children, and this affirms the value of the routine use of PCVs as a public health intervention in children.

## Supplementary Data

Supplementary materials are available at *Clinical Infectious Diseases* online. Consisting of data provided by the authors to benefit the reader, the posted materials are not copyedited and are the sole responsibility of the authors, so questions or comments should be addressed to the corresponding author.

ciaa614_suppl_Supplement_Materials

## Notes

***Author contributions.*** L. H. O., C. M. T., and D. M. W. conceived of the study. C. B. J., M. T. V., A. R., and C. M. T. participated in data collection. L. H. O., K. S., C. M. T., and D. M. W. designed the analysis plan. K. S., C. M. T., D. M. W., A. N. S., and J. L. W. ran the analyses. K. S., C. M. T., and D. M. W. wrote the first draft of the manuscript. K. S., C. M. T., and D. M. W. created the figures. L. H. O. and K. S. contributed equally to this work. All authors contributed to the revisions and approved of the final draft.

***Acknowledgments.*** The authors thank the members of an ad hoc panel that provided feedback on the preliminary results of this study. This group includes Keith Klugman, Gail Rodgers, Prachi Vora, Claire Broome, Marie Griffin, Jennifer Verani, Jennifer Loo, Cynthia Whitney, Fiona Russell, Maria Knoll, Cattram Nguyen, Jocelyn Chan, Adam Cohen, and Vilma Gawryszewski. The authors also thank Carla Vizzoti who was involved in initial data collection and Cuauhtémoc Ruiz-Matus for his leadership as the head of the Unit of Comprehensive Family Immunization, Department of Family, Gender and Life Course, Pan American Health Organization (PAHO).

***Financial support.*** This work was supported by the Bill & Melinda Gates Foundation (grant numbers OPP1156865 to PAHO and OPP117627 to D. M. W.) and the National Institute of Allergy and Infectious Diseases of the National Institutes of Health (grant number R01AI123208 to D. M. W.).

***Potential conflicts of interest.*** D. M. W. has received consulting fees from Pfizer, Merck, GSK, and Affinivax, and grants from Pfizer. K. S., D. M. W., and J. L. W. report personal fees from PAHO. These entities were not involved in any aspect of the current work. L. H. O. is a staff member of PAHO. All other authors report no potential conflicts of interest. All authors have submitted the ICMJE Form for Disclosure of Potential Conflicts of Interest. Conflicts that the editors consider relevant to the content of the manuscript have been disclosed.
