# Supplementary material for: Declines in Pneumonia Mortality Following the Introduction of Pneumococcal Conjugate Vaccines in Latin American and Caribbean Countries
Source: Clin Infect Dis. 2020 May 25;73(2):306–13. doi: 10.1093/cid/ciaa614 (PMC8516507; doi:10.1093/cid/ciaa614)
Supplement: ciaa614_suppl_Supplement_Materials [file ciaa614_suppl_Supplement_Materials.pdf]

SUPPLEMENTARY MATERIALS

Selection of the countries

Ten countries were selected in the study, including countries eligible for Gavi-support (Guyana, Honduras, Nicaragua), and countries not currently eligible for Gavi-support (Argentina, Brazil, Colombia, the Dominican Republic, Ecuador, Mexico, and Peru). The following criteria were applied to select these countries: 1) availability of national mortality data for a period of ≥4 years prior to PCV introduction; 2) introduction of PCV into the NIP prior to 2013; and 3) at least 240 national annual deaths due to any causes in children <5 years of age. These cutoffs were determined based on simulation studies that evaluated how the number of years in the pre-vaccine period and number of cases per month influenced the accuracy (how close an estimate is to the true impact of vaccine) and precision (uncertainty) of estimated vaccine impact [1, 2].

Vaccine schedule and coverage data

The ten countries used a variety of vaccination schedules, which include a primary series of two doses (at 2 and 4 months of age) or three doses (at 2, 4, and 6 months of age), with or without a booster dose (at 12-18 months of age) (Supplementary Table 1) [3]. Many countries switched schedules during the study period; however, we did not evaluate the effects of changes in schedule nor did we attempt to compare schedules or products between countries. Private use of PCVs prior to introduction into the NIP was generally low and not expected to have a measurable impact.

The coverage of pneumococcal conjugate vaccines (PCVs) for the study period, for the country as a whole; and by geographical regions, by age-groups, and by vaccine dose, were obtained from the national immunization program (NIP) in each Latin American and Caribbean (LAC) country. Complete PCV coverage was calculated considering the number of third doses administered over the number of children younger than 1 year of age in countries using the 3+0 or 3+1 schedule; and the number of third doses administered over the number of children aged 12-23 months of age in countries using the 2+1 schedule.

Reasons for excluding children aged <2 months

Children aged <2 months were excluded from the analyses for multiple reasons. First, it is not expected that PCV will have a direct effect in children in this age group because they are not old enough to be vaccinated and many pneumonia cases in this age group are thought to be caused by maternally-acquired bacteria rather than pneumococcus [4]. Second, we found that countries use different strategies to code causes of death for neonates. Some countries exclusively use the ICD-10 P chapter for neonates, while others use a mix of P codes and other chapters. This coding practice for neonates also changed over time in some countries (Supplementary Figure 1). Third, many of the children included in this age group had a recorded age of exactly zero days, so it was not clear whether these children were truly neonates or misassigned to this age group.

Population data

Population estimates were used for calculation of annual vaccine coverage and mortality rates. In each country, annual population estimates were obtained from available census data. For the inter-census period, population was estimated through arithmetic interpolation between the two known data points from available census. We estimated the annual population of children <5 years of age during the study period, and for each of the following age groups: <2, 2-11, 12-23, and 24-59 months of age.

Data management and preliminary analyses

Most countries in the LAC region have publicly funded healthcare systems which covers a large proportion of its population. All data obtained were converted into Excel (.xls) files, using standardized data templates which were elaborated for use in each country in order to facilitate data aggregation and analysis. In each county, descriptive analysis of vaccine coverage, population and mortality data were assessed for completeness, quality and consistency. Preliminary mortality data analysis included absolute and proportional all-cause and pneumonia mortality by year, and age group. PCV use in high risk groups or availability of the vaccine in the private sector was not considered as these groups are very small related to the national population in LAC countries.

Synthetic control models and STL+PCA models

The synthetic control (SC) models and STL+PCA models were fit to the data from the pre-vaccine period, and then the observed values for the control diseases for the post-vaccine period were used by the model to predict

the number of pneumonia deaths that would have been expected if PCV was not introduced. By comparing the observed number of pneumonia deaths in the post-vaccine period to the expected number of cases, we obtain an estimate of the vaccine effect. We calculated rate ratios (which is the cumulative number of observed pneumonia deaths divided by the estimated number of pneumonia deaths in the post-vaccine period) to report the impact of PCV. For these estimates, we report the median and 95% credible intervals (credible intervals, rather than confidence intervals, are used in Bayesian analyses to report uncertainty).

The major assumptions of these models are 1) that the association between pneumonia deaths and the control causes of death is stable over time (e.g., not influenced by other disease-specific interventions, 2) that PCV does not affect the control conditions, and 3) that the control conditions share important causal factors with pneumonia deaths and thus are able to effectively adjust for trends unrelated to PCV [1, 2]. We excluded control causes of death from models if they were likely influenced by PCV and/or if their relationships with pneumonia deaths likely changed over time, based on informal survey of experts in the field (Supplementary Table 2). For example, A00-A09 (rotavirus enteritis) was excluded from the list of control causes of death because countries introduced rotavirus vaccines during our study period, which changed a relationship between rotavirus enteritis and pneumonia deaths over time. Seasonality of the outcome is controlled for by monthly dummy variables.

The SC model uses a Bayesian variable selection method to give more weight to control causes of death that are most correlated with pneumonia deaths in the pre-vaccine period. Spike and slab priors were used for this step [2, 5]. We use minimally-informative prior distributions for all parameters.

In settings where no control causes of death were identified in the SC analysis due to data sparsity, we instead used estimate from the 'STL+PCA' approach [1]. In this method, instead of using a large number of control causes of death to adjust for unrelated trends, we used a single consensus trend of the pre-smoothed versions of the control causes of death and used this in the regression model to adjust for unrelated changes in pneumonia deaths. We present the results from each model separately in Supplementary Table 3 for comparison.

Average estimated total deaths averted per 100,000 population per year

In Supplementary Table1, average estimated total deaths averted per 100,000 population per year was calculated as follows:

$$\frac{Cumulative\ number\ of\ deaths\ averted\ in\ the\ post\ vaccine\ period}{Sum\ of\ annual\ population\ in\ the\ post\ vaccine\ period} * 100{,}000$$

For the denominator, if there were <12 months in a given year, we adjusted the annual population for the number of months included (E.g., if there were only 4 months, we multiplied the annual population by 4/12.)

Review of previous literature

To date, limited real-world evidence of the impact of PCVs on mortality has been available. In Latin American and Caribbean (LAC) countries, previous studies have estimated the individual-level effectiveness on mortality in vaccinated individuals or the overall impact of routine PCV vaccination on mortality rates. Studies have evaluated effects on all-cause mortality, meningitis mortality, and pneumonia mortality. For all-cause mortality, Diaz *et al.* estimated PCV10 effectiveness at 34.8% (95% confidence interval (CI): 23.7%-44.3%) in a nested case control study [6]. Becker-Dreps estimated declines in infant all-cause mortality following introduction of PCV13 at 33% (95% CI: 20%-43%), acknowledging that the number of pneumonia related deaths was too small to explain the reported reduction in infant mortality (138/10,000 child-years) [7]. Two studies addressing mortality due to pneumococcal meningitis reported similar high effectiveness ranging from 65-77.3% in children aged <12 months to 56.8-68.4% in children aged 12-23 months [8, 9].

In general, the few studies that evaluated pneumonia-specific mortality showed substantial declines in mortality rates after PCV introduction but some of them reported wide uncertainty [6, 10-13]. Schuck-Paim *et al* reported significant reductions in childhood pneumonia mortality in children <5 years of age in Brazil, but only in resource-poor municipalities [13].

SUPPLEMENTARY TABLES

Supplementary Table 1. Descriptive statistics for ten Latin American and Caribbean countries.

Country	Population of children <5 years of age in 2010 (millions)	Product, schedule, and year of universal introduction	Vaccine coverage (% with at least 3 doses)[a]	Pre-PCV period	Post-PCV period	Average annual reported incidence of all-cause pneumonia deaths among 2-59 months of age per 100,000 in the pre-PCV period	Average annual reported incidence of all-cause pneumonia deaths among 2-59 months of age per 100,000 in the post-PCV period
Argentina	3.4	PCV13 (2+1) in Jan-2012	85.3	Jan-2005 to Dec-2011	Jan-2012 to Dec-2015	7.8	5.6
Brazil	15.2	PCV10 (3+1) in Mar-2010<br>PCV10 (2+1) in Jan-2017	81.7	Jan-2005 to Feb-2010	Mar-2010 to Dec-2015	15.4	12.3
Colombia	4.5	PCV10 (2+1) in Nov-2011	84	Jan-2005 to Oct-2011	Nov-2011 to Dec-2015	14.2	9.6
Dominican Republic	1.1	PCV13 (2+1) in Sep-2013	NA[b]	Jan-2005 to Aug-2013	Sep-2013 to Dec-2015	8.9	4.6
Ecuador	1.6	PCV7 in Aug-2010<br>PCV10 (2+1) in Feb-2011<br>PCV10 (3+0) in Feb-2014	94.7	Jan-2005 to Jul-2010	Aug-2010 to Dec-2016	27.7	17.8
Guyana	0.1	PCV13 (3+0) in Jan-2011	90	Jan-2000 to Dec-2010	Jan-2011 to Dec-2013	25.2	19.6
Honduras	1.0	PCV13 (3+0) in Jan-2011	88	Jan-2007 to Dec-2010	Jan-2011 to Dec-2016	24.7	26.6
Mexico	11.6	PCV7 (2+1) in Feb-2008 PCV13 (2+1) in Feb-2011[c]	92	Jan-2000 to Jan-2008	Feb-2008 to Dec-2016	18.8	12.3
Nicaragua	0.7	PCV13 (3+0) in Jan-2012	91	Jan-2005 to Dec-2011	Jan-2012 to Dec-2015	27.3	26.4
Peru	2.9	PCV7 (2+1) in Aug-2009<br>PCV10 (2+1) in Dec-2011	82	Jan-2005 to Jul-2009	Aug-2009 to Dec-2014	29.6	21.0

[a] National-level coverage one year after PCV was introduced.
[b] The Dominican Republic prioritized administration of two doses due to a short-term shortage of vaccine supply, so had low uptake of three doses. The second dose coverage ranged from 49·5-92·2% at the subnational level.
[c] Phased introduction starting in 2006 and switch in schedule.
Population aged under 5 was obtained from the Human Development Reports from the United Nations Development Programme (http://hdr.undp.org/en/content/population-aged-under-5-millions).
Abbreviations: PCV, pneumococcal conjugate vaccine; CrI, credible interval.

Supplementary Table 2. List of control causes of death included in the synthetic control model and STL+PCA model.

ICD-10	Description	Exclusions
A10-B99	Certain infectious and parasitic diseases, except intestinal	A40, A49, B95.3
A50-A64	Infections with a predominantly sexual mode of transmission	
A80-A89	Viral and prion infections of the central nervous system	
A90-A99	Arthropod-borne viral fevers and viral hemorrhagic fevers	
B00-B09	Viral infections characterized by skin and mucous membrane lesions	
B15-B19	Viral hepatitis	
B20	Human immunodeficiency virus [HIV] disease	
B35-B49	Mycoses	
B50-B64	Protozoal diseases	
B65-B83	Helminthiases	
C00-C96	Malignant neoplasms	
C00-D48	Neoplasms	
D37-D48	Neoplasms of uncertain behavior, polycythemia vera and myelodysplastic syndromes	
D50-D53	Nutritional anemias	
D55-D59	Hemolytic anemias	
D60-D64	Aplastic and other anemias and other bone marrow failure syndromes	
D65-D69	Coagulation defects, purpura and other hemorrhagic conditions	
D70-D77	Other disorders of blood and blood-forming organs	
E00-E89	Endocrine, nutritional and metabolic diseases	
E10-E13	Type 1, 2 and other specified diabetes mellitus	
E40-E46	Malnutrition	
E70-E89	Metabolic disorders	
G40-G47	Episodic and paroxysmal disorders	
G60-G64	Polyneuropathies and other disorders of the peripheral nervous system	
G80-G83	Cerebral palsy and other paralytic syndromes	
G90-G99	ther disorders of the nervous system	
I00-I99	Diseases of the circulatory system	
I26-I28	Pulmonary heart disease and diseases of pulmonary circulation	
I30-I52	Other forms of heart disease	
I60-I69	Cerebrovascular diseases	

K20-K31	Diseases of esophagus, stomach and duodenum	
K40-K46	Hernia	
K50-K52	Noninfective enteritis and colitis	
K55-K63	Other diseases of intestines	
K65-K67	Diseases of peritoneum and retroperitoneum	
K70-K77	Diseases of liver	
K80-K87	Disorders of gallbladder, biliary tract and pancreas	
K90-K93	Other diseases of the digestive system	
L00-L08	Infections of the skin and subcutaneous tissue	
L00-L99	Diseases of the skin and subcutaneous tissue	
N00-N08	Glomerular diseases	
N00-N99	Diseases of the genitourinary system	
N17-N19	Acute kidney failure and chronic kidney disease	
N30-N39	Other diseases of the urinary system	
P00-P04	Newborn affected by maternal factors and by complications of pregnancy, labor, and delivery	
P00-P96	Certain conditions originating in the perinatal period	
P05-P08	Disorders of newborn related to length of gestation and fetal growth	
P10-P15	Birth trauma	
P20-P29	Respiratory and cardiovascular disorders specific to the perinatal period	P19
P35-P39	Infections specific to the perinatal period	
P50-P61	Hemorrhagic and hematological disorders of newborn	
P70-P74	Transitory endocrine and metabolic disorders specific to newborn	
P76-P78	Digestive system disorders of newborn	
P80-P83	Conditions involving the integument and temperature regulation of newborn	
P90-P96	Other disorders originating in the perinatal period	
Q00-Q07	ongenital malformations of the nervous system	
Q00-Q99	Congenital malformations, deformations and chromosomal abnormalities	
Q20-Q28	Congenital malformations of the circulatory system	
Q30-Q34	Congenital malformations of the respiratory system	
Q35-Q37	Cleft lip and cleft palate	
Q38-Q45	Other congenital malformations of the digestive system	
Q60-Q64	Congenital malformations of the urinary system	
Q65-Q79	Congenital malformations and deformations of the musculoskeletal system	

Q80-Q89	Other congenital malformations	
Q90-Q99	Chromosomal abnormalities, not elsewhere classified	
R00-R09	Symptoms and signs involving the circulatory and respiratory systems	
R00-R99	Symptoms, signs and abnormal clinical and laboratory findings, not elsewhere classified	R65.2
R10-R19	Symptoms and signs involving the digestive system and abdomen	
R50-R69	General symptoms and signs	R65.2
R97-R99	Abnormal tumor markers and Ill-defined and unknown cause of mortality	
V00-X58	Subset of external causes of morbidity	
V00-Y99	External causes of morbidity	
X92-Y09	Assault	
Y21-Y99	Subset of external causes of morbidity	

Abbreviations: ICD-10, International Classification of Diseases 10th edition.

Supplementary Table 3. Rate ratios estimated by synthetic control model, time-trend model, and STL+PCA model by age group in ten Latin American and Caribbean countries.

Age group	Country	Estimated rate ratios (95% CrI)		
		**Synthetic control model**	**Time-trend model**	**STL+PCA model**
2-11m	Argentina	**0.84 (0.72, 0.99)**	1.05 (0.82, 1.37)	1.04 (0.8, 1.35)
	Brazil	**0.92 (0.86, 0.98)**	0.97 (0.87, 1.09)	1.1 (0.99, 1.22)
	Colombia	**0.86 (0.65, 1.05)**	1.2 (0.94, 1.55)	1.93 (1.22, 2.99)
	Dominican Rep.	**0.53 (0.33, 0.93)**	0.65 (0.37, 1.16)	0.67 (0.46, 1.03)
	Ecuador	0.62 (0.56, 0.85)	0.79 (0.57, 1.1)	**0.97 (0.73, 1.3)**
	Guyana	**0.73 (0.4, 1.41)**	1.33 (0.67, 3.43)	1.41 (0.77, 3)
	Honduras	**0.97 (0.55, 1.33)**	0.57 (0.12, 1.72)	0.69 (0.29, 1.19)
	Mexico	**0.78 (0.73, 0.85)**	0.88 (0.78, 0.99)	0.94 (0.83, 1.07)
	Nicaragua	**0.98 (0.8, 1.2)**	0.95 (0.62, 1.42)	0.85 (0.67, 1.07)
	Peru	**0.64 (0.56, 0.83)**	0.87 (0.65, 1.14)	1.46 (0.92, 2.21)
12-23m	Argentina	**0.65 (0.5, 0.85)**	0.62 (0.38, 1.02)	0.73 (0.47, 1.15)
	Brazil	**0.91 (0.81, 1.03)**	1.12 (0.91, 1.39)	1.09 (0.92, 1.3)
	Colombia	**0.6 (0.52, 0.73)**	0.9 (0.67, 1.22)	0.89 (0.69, 1.15)
	Dominican Rep.	**0.75 (0.38, 1.71)**	0.8 (0.28, 2.4)	6 (1.2, Inf*)
	Ecuador	**0.73 (0.57, 0.91)**	0.88 (0.6, 1.28)	0.97 (0.71, 1.33)
	Mexico	**0.97 (0.85, 1.11)**	1.11 (0.92, 1.35)	1.1 (0.89, 1.35)
	Peru	**0.6 (0.53, 0.78)**	0.8 (0.55, 1.16)	0.9 (0.69, 1.18)
24-59m	Argentina	**0.99 (0.8, 1.27)**	0.9 (0.59, 1.41)	0.97 (0.73, 1.28)
	Brazil	0.82 (0.75, 0.92)	0.93 (0.72, 1.19)	**0.93 (0.75, 1.14)**
	Colombia	0.79 (0.67, 0.97)	0.9 (0.63, 1.3)	**0.9 (0.64, 1.27)**
	Dominican Rep.	**0.82 (0.41, 2)**	1.4 (0.58, 7)	3.5 (0.37, Inf*)
	Ecuador	0.63 (0.55, 0.73)	0.8 (0.53, 1.2)	**0.58 (0.29, 1.1)**
	Guyana	1 (0.25, Inf*)	1 (0.07, Inf*)	**2 (0.12, Inf*)**
	Mexico	**1.12 (0.99, 1.26)**	1.07 (0.84, 1.35)	1.36 (0.94, 1.94)
	Nicaragua	0.83 (0.65, 1.1)	0.71 (0.38, 1.33)	**0.72 (0.46, 1.03)**
	Peru	0.79 (0.66, 0.91)	0.82 (0.52, 1.26)	**0.95 (0.68, 1.3)**
2-59m	Argentina	**0.92 (0.74, 1.11)**	0.94 (0.75, 1.18)	0.94 (0.76, 1.16)
	Brazil	**0.98 (0.92, 1.04)**	1.01 (0.92, 1.11)	1.18 (1.06, 1.32)
	Colombia	**0.76 (0.65, 0.97)**	1.1 (0.9, 1.35)	1.47 (1.09, 1.96)

Dominican Rep.	**0.9 (0.53, 1.5)**	0.73 (0.49, 1.12)	1.34 (0.9, 2.1)
Ecuador	**0.75 (0.59, 0.96)**	0.85 (0.68, 1.04)	0.85 (0.71, 1)
Guyana	0.75 (0.43, 1.42)	1.23 (0.63, 2.7)	**1 (0.61, 1.93)**
Honduras	**1.16 (0.77, 1.5)**	0.43 (0.14, 1.11)	0.73 (0.38, 1.21)
Mexico	**0.89 (0.82, 0.97)**	0.97 (0.87, 1.08)	1.08 (0.94, 1.23)
Nicaragua	0.91 (0.78, 1.07)	0.92 (0.66, 1.3)	**0.81 (0.67, 1)**
Peru	**0.65 (0.59, 0.8)**	0.86 (0.69, 1.08)	0.92 (0.78, 1.08)

Abbreviations: m, months; CrI, credible interval; Inf, infinity.

Estimated rate ratios that we reported as final results are in bold. We reported estimates generated by synthetic control models in most settings except in strata because this model outperforms other methods in adjusting for underlying trends in the data. Where the synthetic control models were unable to detect an adequate control time series due to data sparsity, estimates generated by the STL+PCA model were reported as final results.

*Upper bounds of the 95% CrIs are infinity, because the denominator of the rate ratio, which is the cumulative number of counterfactual pneumonia mortality, was zero.

Supplementary Table 4. Top three control causes of death selected by the Bayesian variable selection method in the synthetic control models and estimated impact of PCV after excluding these top three control causes from the models (children 2-59 months of age in ten Latin American and Caribbean countries).

Country	RR estimated by the full SC model	Top 1 control cause of death (inclusion probability)	RR estimated by the SC model without the top 1 control (95% CrI)	Top 2 control cause of death (inclusion probability)	RR estimated by the SC model without the top 1 and 2 controls (95% CrI)	Top 3 control cause of death (inclusion probability)	RR estimated by the SC model without the top 1-3 controls (95% CrI)
Argentina	0.92 (0.74, 1.11)	All cause mortality (0.39)	0.91 (0.73, 1.14)	A00-B99 (0.31)	0.84 (0.71, 1.02)	R95-R99 (0.18)	0.83 (0.71, 1)
Brazil	0.98 (0.92, 1.04)	All cause mortality (1.00)	0.91 (0.85, 1.04)	Q60-Q64 (0.13)	0.91 (0.85, 1.03)	G60-G64 (0.01)	0.91 (0.85, 1.04)
Colombia	0.76 (0.65, 0.97)	A00-B99 (0.7)	0.85 (0.65, 1.02)	N00-N99 (0.32)	0.86 (0.69, 1.03)	V01-X59 (0.31)	0.84 (0.62, 1.01)
Dominican Rep.	0.9 (0.53, 1.5)	Q00-Q99 (0.72)	0.68 (0.45, 1.03)	A00-B99 (0.61)	0.68 (0.4, 1.11)	P00-P96 (0.38)	0.64 (0.37, 1.03)
Ecuador	0.75 (0.59, 0.96)	R00-R09 (0.41)	0.68 (0.58, 0.84)	R00-R99 (0.22)	0.66 (0.58, 0.8)	Q00-Q99 (0.16)	0.64 (0.58, 0.78)
Guyana	1 (0.61, 1.93)	P35-P39 (0.19)	0.71 (0.42, 1.35)	All cause mortality (0.16)	0.73 (0.42, 1.42)	B20-B24 (0.14)	0.69 (0.41, 1.29)
Honduras	1.16 (0.77, 1.5)	Q20_Q28 (0.89)	1.06 (0.65, 1.42)	C00-D48 (0.79)	1.02 (0.62, 1.38)	Q00-Q99 (0.67)	1.01 (0.59, 1.38)
Mexico	0.89 (0.82, 0.97)	All cause mortality (1.00)	0.89 (0.8, 1)	Q90_Q99 (0.19)	0.9 (0.81, 1)	E40-E46 (0.11)	0.91 (0.82, 1.01)
Nicaragua	0.81 (0.67, 1)	A00-B99 (0.11)	0.92 (0.78, 1.07)	E00-E90 (0.10)	0.92 (0.79, 1.07)	E40-E46 (0.08)	0.92 (0.79, 1.07)
Peru	0.65 (0.59, 0.8)	R00-R09 (0.56)	0.68 (0.6, 0.83)	G90-G99 (0.18)	0.67 (0.6, 0.81)	N00-N99 (0.13)	0.66 (0.59, 0.84)

All-cause mortality does not include deaths caused by respiratory illness (i.e., J chapters of the ICD-10 code). Inclusion probabilities show how often these control causes were selected in the Bayesian variable selection method.

Abbreviations: PCV, pneumococcal conjugate vaccine; RR, rate ratio; SC, synthetic control; CrI, credible interval; ICD-10, International Classification of Diseases 10th edition.

Supplementary Table 5. Estimated impact of PCV and deaths averted by age group in ten Latin American and Caribbean countries.

Age group	Country	Estimated rate ratio (95% CrI)	Estimated total deaths averted since PCV introduction (95% CrI)	Estimated total deaths averted per 100,000 population since PCV introduction (95% CrI)
2-11m	Argentina	0.84 (0.72, 0.99)	127 (34, 225)	5 (1.3, 8.8)
	Brazil	0.92 (0.86, 0.98)	538 (176, 908)	3.2 (1, 5.4)
	Colombia	0.86 (0.65, 1.05)	144 (-35, 464)	4.8 (-1.2, 15.4)
	Dominican Rep.	0.53 (0.33, 0.92)	51 (7, 118)	12.9 (1.8, 29.8)
	Ecuador	0.97 (0.73, 1.3)	42 (-203, 371)	2.3 (-11.3, 20.6)
	Guyana	0.73 (0.4, 1.41)	15 (-7, 48)	41.3 (-19.3, 132.2)
	Honduras	0.97 (0.55, 1.33)	73 (-226, 959)	8.2 (-25.4, 107.6)
	Mexico	0.78 (0.73, 0.85)	2171 (1391, 2952)	13 (8.3, 17.7)
	Nicaragua	0.98 (0.8, 1.2)	-13 (-92, 79)	-2.8 (-20, 17.2)
	Peru	0.64 (0.56, 0.83)	972 (412, 1389)	36.8 (15.6, 52.6)
12-23m	Argentina	0.65 (0.5, 0.85)	75 (34, 129)	2.5 (1.1, 4.3)
	Brazil	0.91 (0.81, 1.03)	232 (-6, 482)	1.2 (0, 2.4)
	Colombia	0.6 (0.52, 0.73)	200 (111, 275)	5.6 (3.1, 7.6)
	Dominican Rep.	0.75 (0.38, 1.71)	4 (-7, 24)	0.8 (-1.5, 5.1)
	Ecuador	0.73 (0.57, 0.91)	158 (49, 305)	8.7 (2.7, 16.9)
	Mexico	0.97 (0.85, 1.11)	95 (-160, 357)	0.5 (-0.8, 1.8)
	Peru	0.6 (0.53, 0.78)	327 (137, 447)	10.4 (4.3, 14.2)
24-59m	Argentina	0.99 (0.8, 1.27)	3 (-29, 37)	0 (-0.3, 0.4)
	Brazil	0.93 (0.75, 1.14)	183 (-187, 653)	0.3 (-0.3, 1.1)
	Colombia	0.9 (0.64, 1.27)	37 (-45, 145)	0.3 (-0.4, 1.4)
	Dominican Rep.	0.82 (0.41, 2)	9 (-4, 31)	0.6 (-0.3, 2.2)
	Ecuador	0.58 (0.29, 1.1)	266 (-22, 837)	3.9 (-0.3, 12.3)
	Guyana	2 (0.12, Inf*)	-1 (-3, 18)	-0.8 (-2.4, 14.3)
	Mexico	1.12 (0.99, 1.26)	-130 (-280, 44)	-0.2 (-0.5, 0.1)
	Nicaragua	0.72 (0.46, 1.03)	29 (0, 73)	1.8 (0, 4.4)
	Peru	0.95 (0.68, 1.3)	35 (-106, 235)	0.4 (-1.1, 2.5)

Abbreviations: m, months; CrI, credible interval; Inf, infinity; PCV, pneumococcal conjugate vaccine.
*Upper bounds of the 95% CrIs for <2 months of age and 24-59 months of age in Guyana are infinity, because the denominator of the rate ratio, which is the cumulative number of counterfactual pneumonia mortality, was zero.

Supplementary Table 6. Estimated impact of PCV among children 2-59 months of age in four Latin American countries, using a definition of all-cause pneumonia that searched for the relevant ICD-10 codes anywhere in the record.

**Country**	**Estimated rate ratio (95% CrI)**
Brazil	0.95 (0.91, 0.99)
Colombia	0.77 (0.69, 0.91)
Honduras	1.11 (0.83, 1.35)
Peru	0.98 (0.84, 1.15)

Abbreviations: CrI, credible interval; ICD-10, International Classification of Diseases 10th edition; PCV, pneumococcal conjugate vaccine.
Other countries were not included in this sensitivity analysis as the non-primary causes of death were not available.

Supplementary Table 7. Estimated impact of PCV and deaths averted among children 2-59 months of age in ten Latin American and Caribbean countries, using two-year transition period.

Country	Estimated rate ratio (95% CrI)
Argentina	0.92 (0.71, 1.17)
Brazil	0.96 (0.91, 1.02)
Colombia	0.74 (0.62, 0.95)
Dominican Rep.	1.08 (0.58, 2.17)
Ecuador	0.73 (0.56, 0.98)
Guyana	1.06 (0.49, 2.83)
Honduras	1.11 (0.66, 1.49)
Mexico	0.89 (0.82, 0.98)
Nicaragua	0.76 (0.61, 0.95)
Peru	0.63 (0.57, 0.81)

Abbreviations: PCV, pneumococcal conjugate vaccine; CrI, credible interval.

SUPPLEMENTARY FIGURES

Supplementary Figure 1. Changes in the use of J12-J18 compared to perinatal ICD-10 codes for pneumonia in ten Latin American and Caribbean countries.

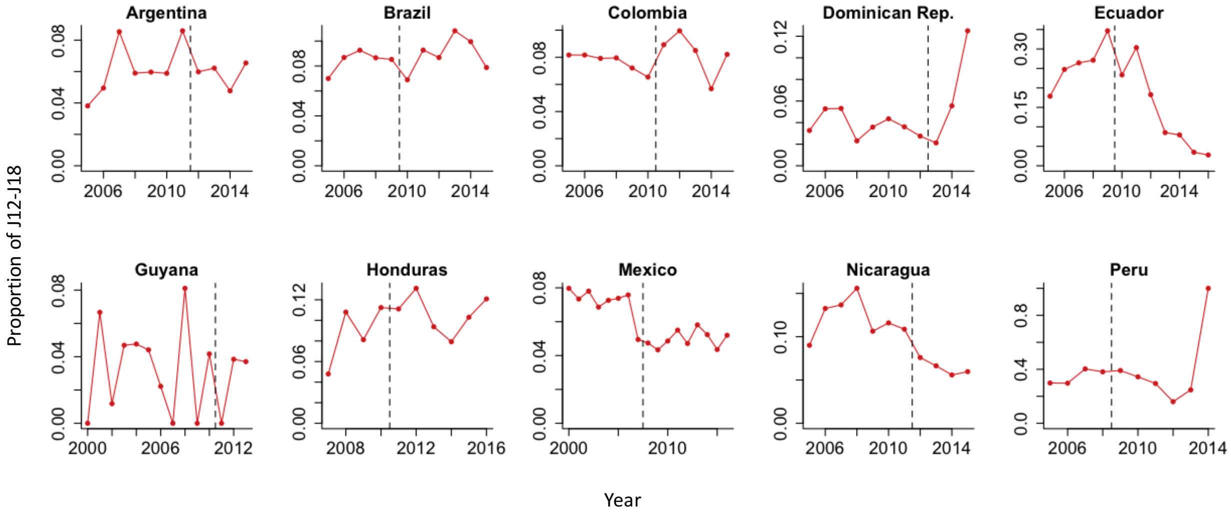

Abbreviation: ICD, Int International Classification of Diseases 10th edition; PCV, pneumococcal conjugate vaccine.

Changes in the use of all-cause pneumonia (J12-J18) compared to perinatal codes (P22: respiratory distress of newborn, and P28: other respiratory conditions originating in the perinatal period) are described by the proportion of J12-J18 which was calculated as follows:

$$\text{Proportion of J12-J18} = \frac{\text{Number of J12-J18}}{\text{Number of J12-J18, P22, and P28}}$$

Vertical dashed lines show the timing of PCV introduction in each country.

Supplementary Figure 2. Standardized quality indicators for mortality data in ten Latin American and Caribbean countries.

A.

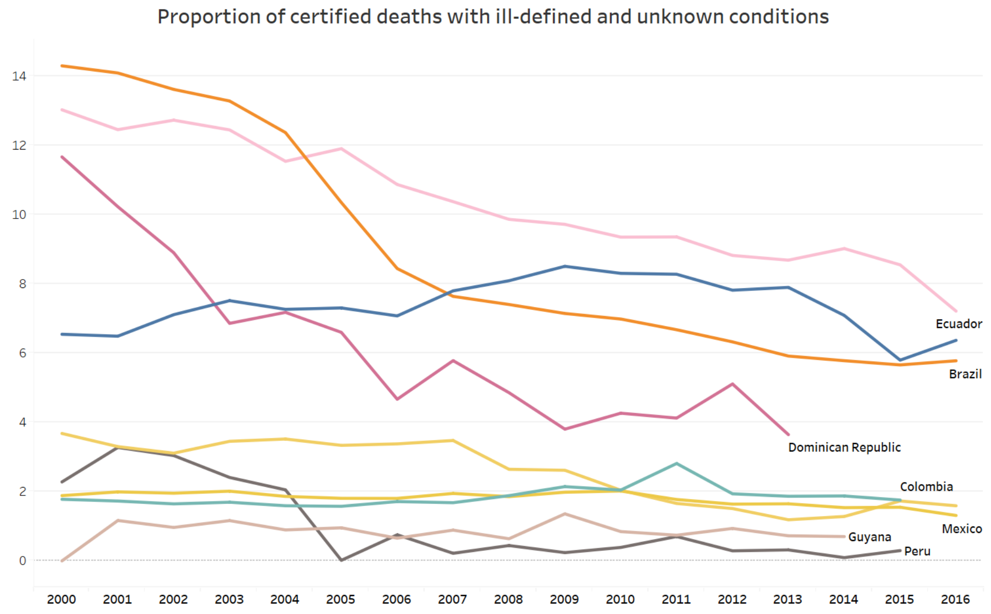

B.

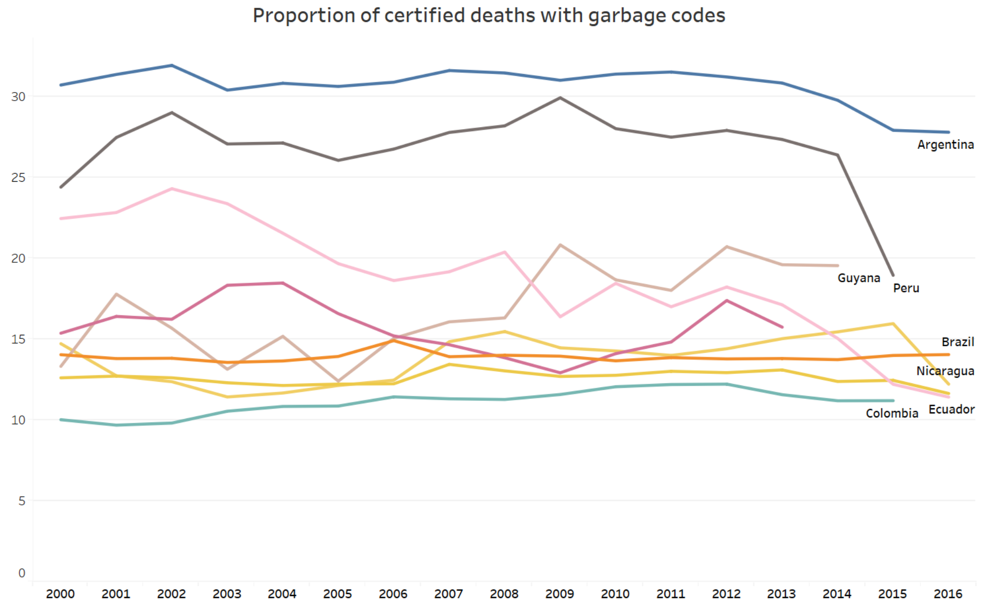

C.

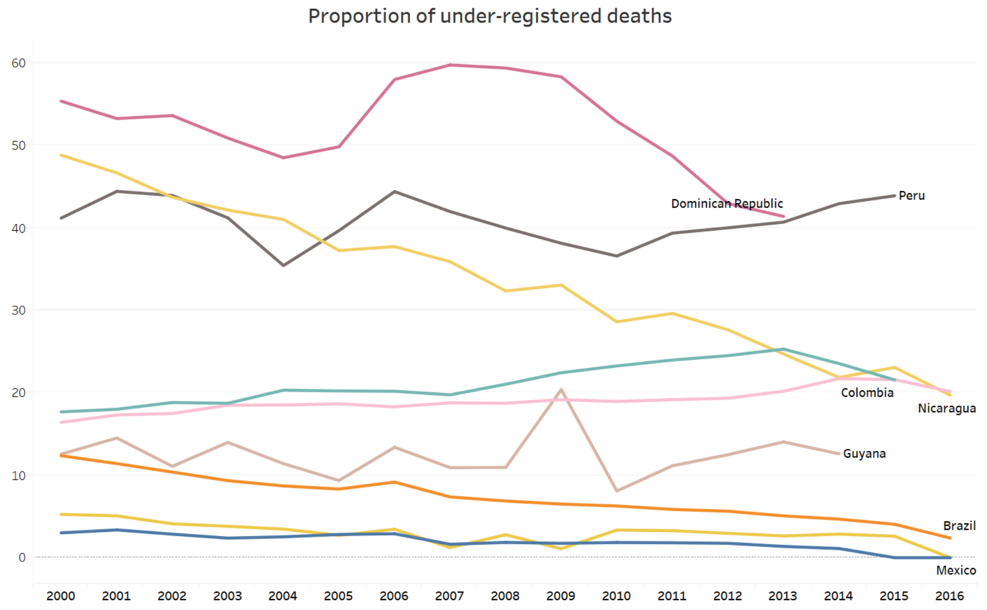

D.

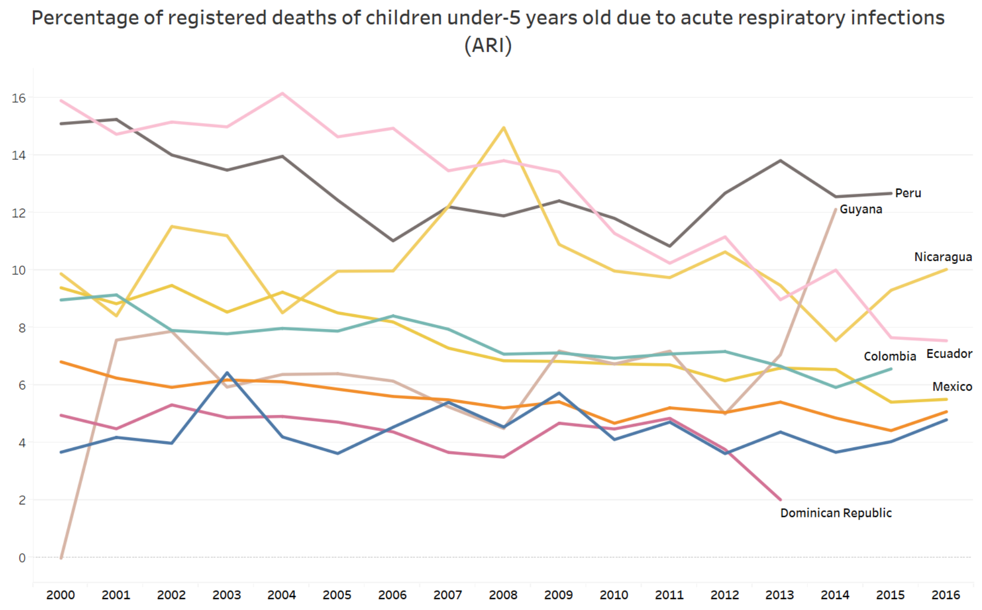

Data from the PLISA dashboard (http://www.paho.org/data/index.php/es/indicadores.html) were used to create these figures.

Supplementary Figure 3. Annual time series for observed and predicted number of pneumonia deaths by age group in ten Latin American and Caribbean countries.

A. 2-11 months of age

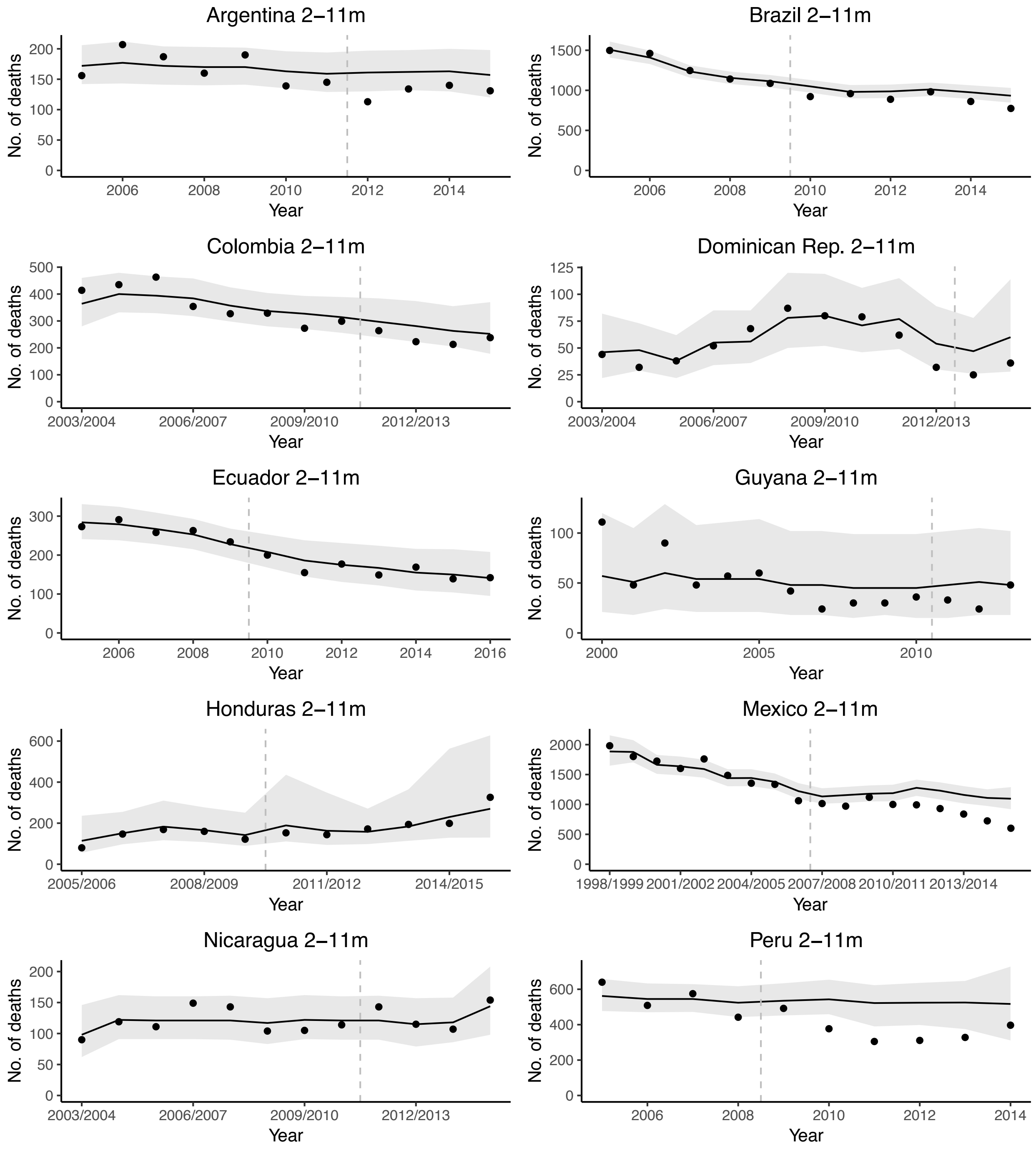

B. 12-23 months of age

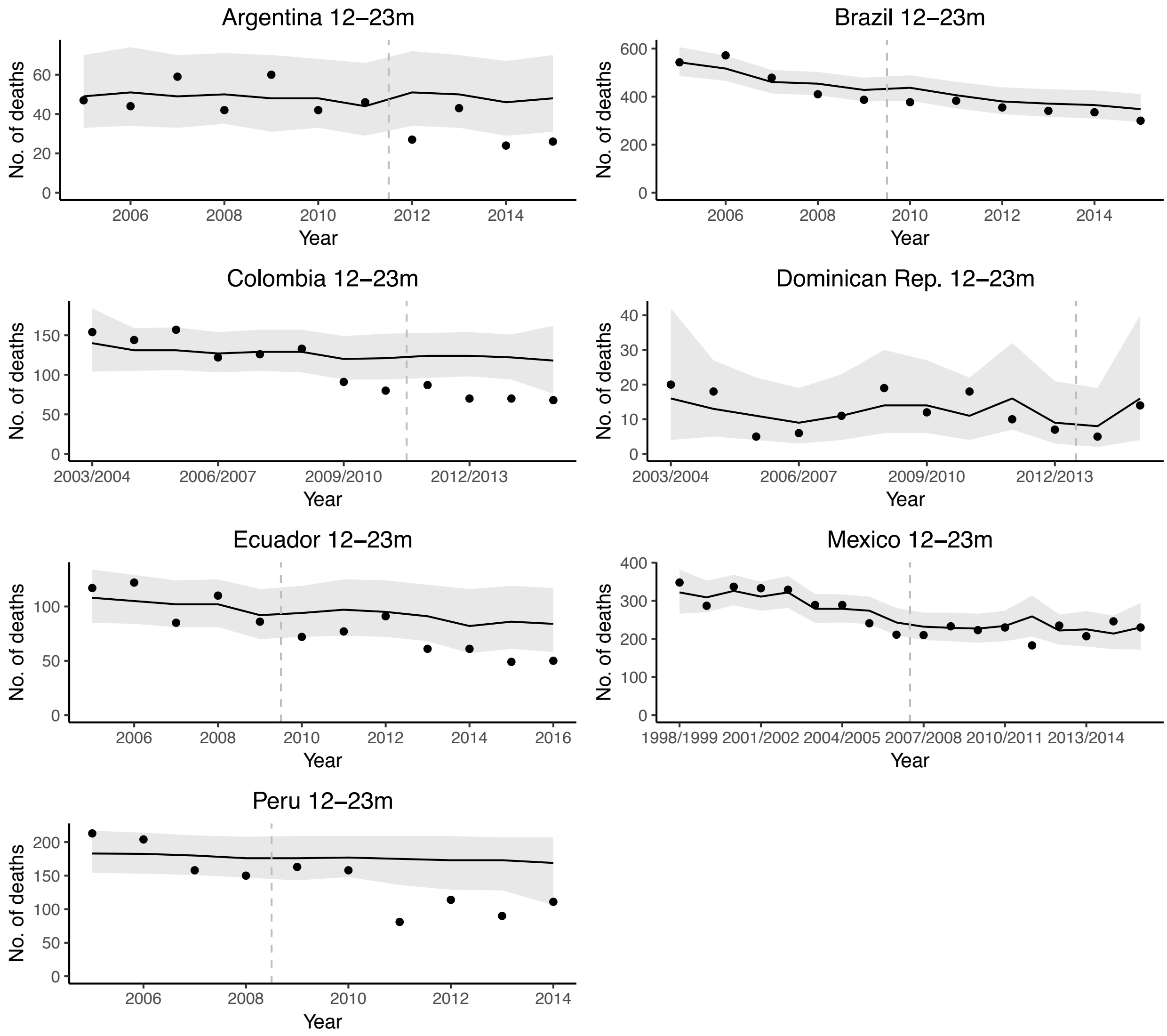

C. 24-59 months of age

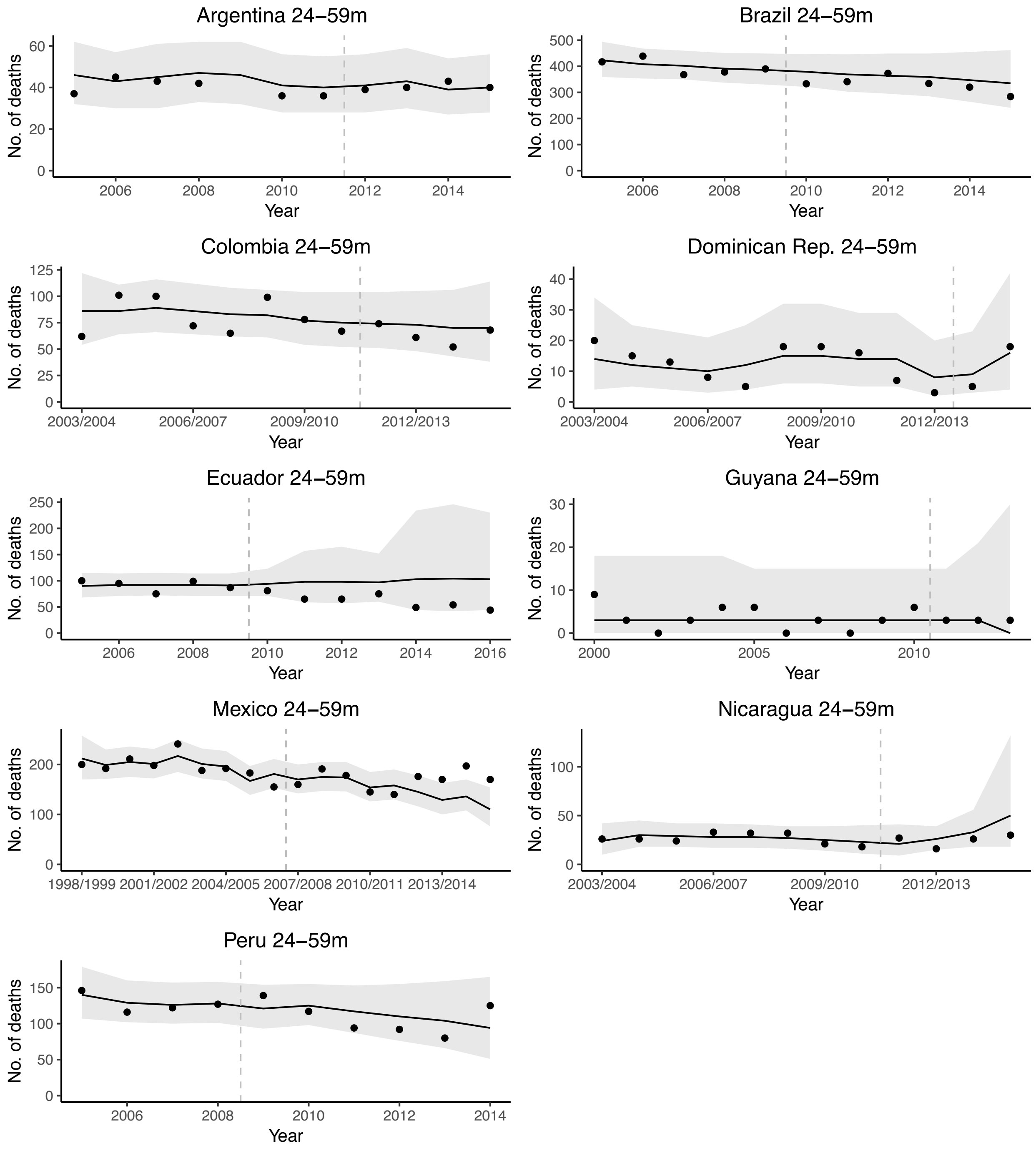

Abbreviation: ICD, Int International Classification of Diseases 10th edition; PCV, pneumococcal conjugate vaccine.
Dots represent the observed number of pneumonia deaths (ICD-10: J12-J18).
Lines and grey-shaded areas represent point estimates and 95% credible intervals of the predicted pneumonia deaths, respectively. Vertical dashed lines show the timing of PCV introduction in each country. Year was defined as from January to December in the countries in the Southern Hemisphere, and from July to June in the countries in the Northern Hemisphere.

REFERENCES

1. Shioda K, Schuck-Paim C, Taylor R, et al. Challenges in Estimating the Impact of Vaccination with Sparse Data. Epidemiology **2019**; 30(1): 61-8.
2. Bruhn CA, Hetterich S, Schuck-Paim C, et al. Estimating the population-level impact of vaccines using synthetic controls. Proceedings of the National Academy of Sciences of the United States of America **2017**; 114(7): 1524-9.
3. World Health Organization. Pneumococcal vaccines WHO position paper--2012. Releve epidemiologique hebdomadaire **2012**; 87(14): 129-44.
4. McIntosh K. Community-acquired pneumonia in children. N Engl J Med **2002**; 346(6): 429-37.
5. Brodersen KH, Gallusser F, Koehler J, Remy N, Scott SL. Inferring causal impact using Bayesian structural time-series models. The Annals of Applied Statistics **2015**; 9(1): 247-74.
6. Diaz J, Terrazas S, Bierrenbach A, et al. Effectiveness of the 10-Valent Pneumococcal Conjugate Vaccine (PCV-10) in Children in Chile: A Nested Case-Control Study Using Nationwide Pneumonia Morbidity and Mortality Surveillance Data. PloS one **2016**; 11(4).
7. Becker-Dreps S, Amaya E, Liu L, et al. Changes in childhood pneumonia and infant mortality rates following introduction of the 13-valent pneumococcal conjugate vaccine in Nicaragua. The Pediatric infectious disease journal **2014**; 33(6): 637-42.
8. Hirose T, Maluf E, Rodrigues C. Pneumococcal meningitis: epidemiological profile pre- and post-introduction of the pneumococcal 10-valent conjugate vaccine. The Journal of pediatrics **2015**; 91(2): 130-5.
9. Grando IM, Moraes C, Flannery B, et al. Impact of 10-valent pneumococcal conjugate vaccine on pneumococcal meningitis in children up to two years of age in Brazil. Cad Saude Publica **2015**; 31(2): 276-84.
10. Suarez V, Michel F, Toscano C, et al. Impact of pneumococcal conjugate vaccine in children morbidity and mortality in Peru: Time series analyses. Vaccine **2016**; 34(39): 4738-43.
11. Kupek E, Vieira I. Impact of PCV10 pneumococcal vaccine on mortality from pneumonia in children less than one year of age in Santa Catarina State, Brazil. Cad Saude Publica **2016**; 32(3): 1.
12. Becker-Dreps S, Blette B, Briceno R, et al. Changes in the incidence of pneumonia, bacterial meningitis, and infant mortality 5 years following introduction of the 13-valent pneumococcal conjugate vaccine in a "3+0" schedule. PloS one **2017**; 12(8): e0183348.
13. Schuck-Paim C, Taylor R, Alonso W, Weinberger D, Simonsen L. Effect of pneumococcal conjugate vaccine introduction on childhood pneumonia mortality in Brazil: a retrospective observational study. Lancet Glob Health **2019**; 7(2): e249-e56.
